# Cumulative Effects of Watershed Disturbances and Run-of-river Dams on Mercury Cycling: Case Study and Recommendations for Environmental Managers

**DOI:** 10.1007/s00267-024-01990-6

**Published:** 2024-05-22

**Authors:** M. Amyot, F. Bilodeau, A. Tremblay, D. Planas, D. Walsh, D. E. Ponton

**Affiliations:** 1 GRIL, Département de Sciences Biologiques, Université de Montréal, 1375 Thérèse-Lavoie-Roux Ave., Montréal, QC H2V 0B3 Canada; 2 Hydro-Québec, Direction Environnement, 800 De Maisonneuve Est Blvd., Montréal, QC H2Z 1A4 Canada; 3 GRIL, GEOTOP, Département de Sciences Biologiques, Université du Québec à Montréal, 141 Président-Kennedy Ave., Montréal, QC H2X 1Y4 Canada; 4 GRIL, Department of Biology, Concordia University, 7141 Sherbrooke St. West, Montréal, QC H4B 1R6 Canada

**Keywords:** Renewable energy, Hydroelectricity, Pollutants, Forest fire, Logging, Metals

## Abstract

Run-of-river power plants (ROR) represent the majority of hydroelectric plants worldwide. Their environmental impacts are not well documented and are believed to be limited, particularly regarding the contamination of food webs by methylmercury (MeHg), a neurotoxin. RORs are typically installed in small rivers where combined effects of watershed disturbances with dam construction can complicate environmental management. We report a multi-year case study on the Saint-Maurice River (Canada) where an unpredicted temporary increase in MeHg accumulation in predator fish was observed after the construction of two ROR plants. The associated pondages acted as sedimentation basins for mercury (Hg) and organic matter from a watershed disturbed by a forest fire and by logging. This fresh organic carbon likely fueled microbial MeHg production. Hg methylation was more associated with environmental conditions than to the presence of Hg, and main methylating microbial groups were identified. A constructed wetland was a site of significant Hg methylation but was not the main source of the fish Hg increase. Organic carbon degradation was the main driver of MeHg accumulation at the base of the food chain whereas trophic levels explained the variations at the top of the food chain. Overall, carbon cycling was a key driver of Hg dynamics in this system, and ROR plants can cause temporary (ca. 12 years) Hg increase in food webs when developed in disturbed watersheds, although this increase is smaller than for large reservoirs. Recommendations for future ROR construction are to establish a good environmental monitoring plan with initial high temporal resolution and to consider recent and potential watershed disturbances in the plan.

## Introduction

It is estimated that there are more than 80,000 small hydropower plants (SHP) in operation or under construction in the world and that this number may triple if all potential generation capacity were to be developed (Couto and Olden 2018). There are approximately 11 SHP for every large hydropower plant. The definition of SHP differs among countries, with values of maximum energy produced generally ranging from 10 to 50 MW, with 70% of countries with formal definition using the value of 10 MW (Couto and Olden 2018). These SHP are seen as important components of energy policies by many governments in order to move towards sustainable, renewable energy (Lange et al. 2019). Small hydropower plants are commonly considered to have less environmental impacts than hydropower plants with large reservoirs, even though there is a scarcity of ecological studies on the impacts of this type of infrastructure (Couto and Olden 2018). In many countries, the degree of pre-assessment depends on the planned installed capacity, which leaves many low-capacity projects without any proper assessment of environmental or socioeconomic impacts (Lange et al. 2019). This is of concern since some evidence suggest a potentially bigger environmental impact of SHP per megawatt of electricity produced, compared to large hydropower plants, due to the cumulative impact of small dams over space and time (Lange et al. 2019; Ziv et al. 2012).

There is a great diversity of SHP, which can be classified according to the level of flow control (with or without storage) and the presence of diversion structure (Couto and Olden 2018; McManamay et al. 2016). Among those, run-of-river power plants (ROR) are characterized by no or limited storage, in which case the storage reservoir is referred to as pondage. RORs may alter the natural flow regime of a river and impact aquatic ecosystems at different trophic levels (Kuriqi et al. 2021). Run-of-river power plant types include dam-toe, diversion weir and pondage schemes. According to a recent global-scale analysis, the latter two are considered to be less environmentally-friendly (Kuriqi et al. 2021) with potential ecological impacts including water depletion downstream of the diversion, water quality deterioration, loss of longitudinal connectivity, habitat degradation and simplification of the biota community composition (Kuriqi et al. 2021).

One important environmental and socio-economic consequence of hydroelectricity is the observed increase in mercury (Hg) contamination of food webs and predatory fish used for consumption by communities. This issue is very well documented for large boreal hydroelectric plants with vast reservoirs (Bilodeau et al. 2017). In these instances, large quantities of terrestrial organic matter are flooded thereby creating zones where microbes can methylate inorganic mercury into the strong neurotoxicant methylmercury (MeHg) that is then bioaccumulated by organisms and biomagnified along the food web. Flooded soils are prone to Hg methylation because they provide fresh organic matter and reductive conditions that can fuel anaerobic activity by methylating microorganisms (Hall et al. 2005). These large boreal reservoirs are likely more at risk for Hg biomagnification than their tropical counterparts because of efficient trophic transfer along simplified foodwebs (Brinkmann and Rasmussen 2010; Lavoie et al. 2013; Sabo et al. 2009; Vander Zanden et al. 1999). In contrast, because ROR typically do not flood large areas, it is often inferred that they do not significantly modify mercury cycling, since only a limited amount of organic matter and newly-produced MeHg can be transferred to the river, which can rapidly dilute these inputs in its flow. However, there is a scarcity of data on this issue.

A multi-year study conducted on the Saint-Maurice River in Quebec (Canada) has recently reported for the first time that ROR may contribute to unexpected increases of Hg in fish under certain circumstances (Ponton et al. 2021). Here, we consider mercury cycling in this river at different scales, from the genes involved in microbial methylation to large-scale landscape interactions between disturbances such as fire and logging. We explore this case study and its conclusions and combine it with other research reports in the field. We then develop a conceptual model of the combined effects of ROR and watershed disturbances on Hg cycling and provide a series of recommendations for decision-makers from the hydroelectric sector, with a focus on the North American environment. Methodological details are reported in the cited literature.

## The Saint-Maurice River Case Study

**Fig. 1:**
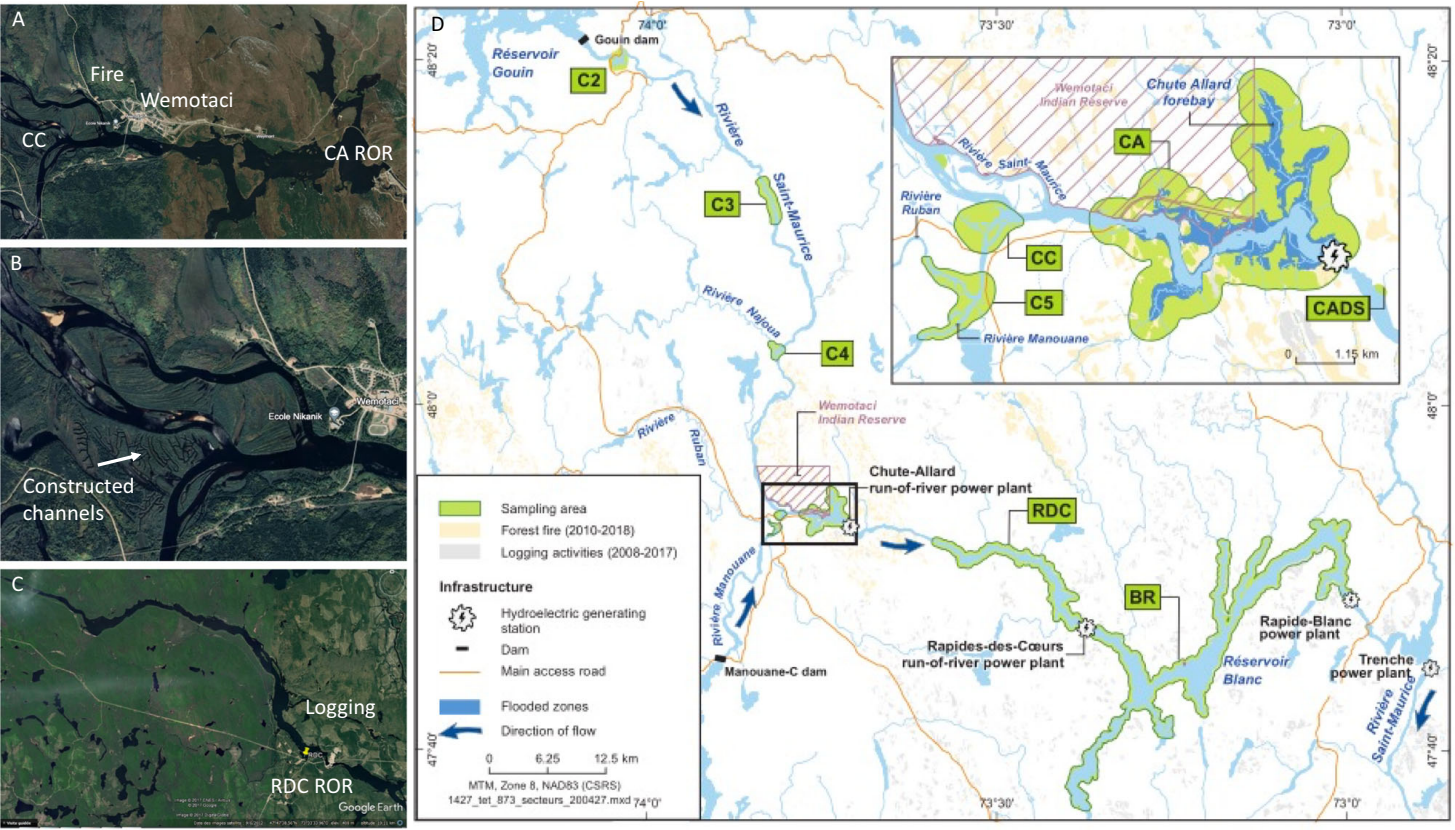
Aerial view of portions of the Saint-Maurice River. **A** Location of Chute-Allard ROR (CA ROR) relative to the Atikamekw community of Wemotaci, to the 2010 forest fire (Fire) and the constructed channels (CC); **B** Aerial view of the constructed channels; **C** Location of Rapides-des-Coeurs ROR (RDC ROR) relative to logging activities (Logging); **D** Map of the region representing the main sampling sites, the areas burned by forest fires and affected by logging activities, adapted from Ponton et al. (2021), copyright 2021 American Chemical Society

The Saint-Maurice River (Quebec, Canada), also known in Atikamekw as the Tapiskwan sipi, is 563 km long and has an average annual flow of 730 m^3^/s at the river mouth. It is a tributary of the St. Lawrence River. At its head is located a large reservoir, Reservoir Gouin (1789 km^2^) which was flooded in 1918. The river is harnessed by an additional reservoir dam (Rapide-Blanc dam, and the Reservoir Blanc, RB) and ten small ROR power plants. Among those ROR, two were constructed in 2008 upstream of Reservoir Blanc, namely Chute-Allard (CA ROR, capacity: 62 MW) and Rapides-des Coeurs (RDC ROR, capacity: 79 MW), near the Atikamekw First Nation Reserve of Wemotaci (Fig. 1).

### Unexpected Surge in Fish Hg Accumulation, Development of Research Partnerships and Research Hypotheses

**Fig. 2:**
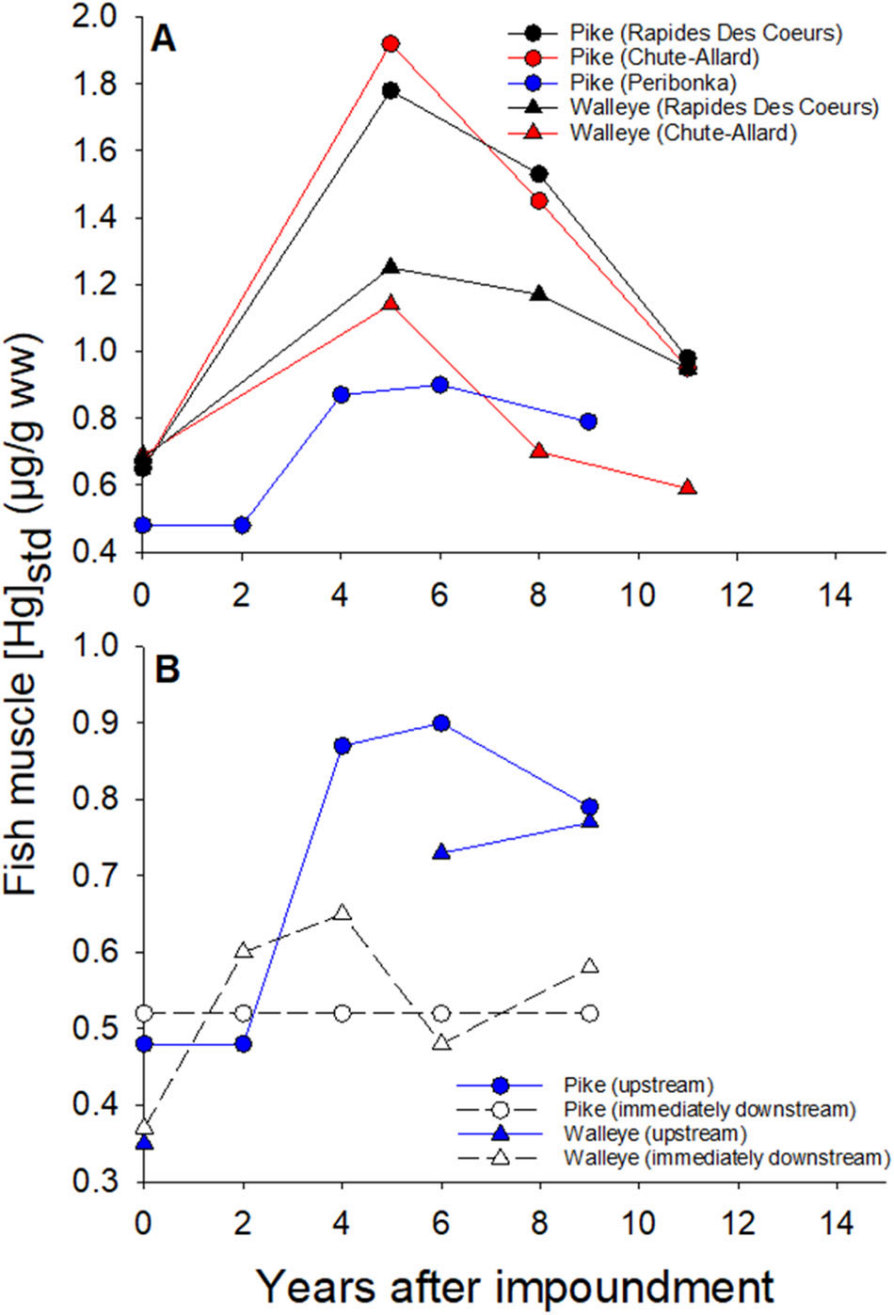
Temporal changes in standardized Hg concentrations (**A**) in the pondages of three run-of-river dams for northern pike (standardized at 600 mm) and walleye (standardized at 400 mm), namely Chute-Allard, Rapides-des-Coeurs on the Saint-Maurice River and Péribonka on the Péribonka River; **B** in the pondage and immediately downstream of the Péribonka dam. Data from (Cossette et al. 2019; Ponton et al. 2021; Sacotte et al. 2020). To obtain fish muscle [Hg]_std_, [THg] was measured in the muscle of 30 individuals from a given fish species; a polynomial regression of total [Hg] as a function of fish length was done from which a standardized [THg] for a given length was calculated ([THg_std_], northern pike: 600 mm, walleye: 400 mm), following (Tremblay et al. 1998). Details on fish sampling size and methods can be found in Supplementary information and in (Ponton et al. 2021)

Pre-construction modeling of the effect of these two recent RORs by Hydro-Québec (the main public electric utility company in Québec) on Hg accumulation in fish indicated that there would be no noticeable change (Hydro-Québec 2004). The models used were developed for large reservoirs and were heavily influenced by the flooded area as a key driver of Hg accumulation in fish. Environmental monitoring of Hg in fish done five years after construction (2013) revealed an unexpected and significant increase of bioaccumulated Hg, particularly in large predatory fish such as walleye (*Sander vitreus*) and northern pike (*Esox Lucius*) (Fig. 2 presented here) (Ponton et al. 2021). This Hg increase triggered a modification of fish consumption advisories for the Indigenous community living nearby. Consequently, a major communication program was deployed, in collaboration with public health agencies, to inform communities and a specific fish consumption guide was produced (Hydro-Québec 2014).

A four-year university-industry partnership was developed to understand what caused this unexpected increase. This partnership was recently renewed and now involves the Indigenous Atikamekw Council of Wemotaci as a full partner. Different hypotheses were considered to explain the unexpected significant increase in Hg in fish. First, as a compensatory measure, a system of constructed channels was developed upstream of Chute-Allard pondage, to compensate for habitat modification and to increase biodiversity. This wetland was constructed by excavating several kilometers of interconnected channels of about four to eight meters wide (Fig. 1B). It is well known that wetlands can be hotspots of MeHg production (Hall et al. 2008; Loseto et al. 2004), since they are often rich in organic matter and may have anoxic zones. It was therefore hypothesized that these constructed channels may behave as MeHg production hotspots for downstream sites. Secondly, the construction of dams along a river system may alter the flux of particulate matter and promote sedimentation upstream from the dams (Ma et al. 2023; Sow et al. 2016). These sediments could then act as sites for MeHg production. Thirdly, the flooding of even a small area could have triggered some level of MeHg production. Here, the construction of CA ROR and RDC ROR flooded 2 and 3.7 km^2^, respectively (Fig. 1A, C). Also, the creation of pondages could have altered the structure of the community, from microbes to fish, in such a way as to increase the transfer of MeHg along the food web. Some studies have indeed reported community changes caused by ROR, but without linking them to contaminant flow across food webs (Baumgartner et al. 2020; Bilotta et al. 2017; Cella-Ribeiro et al. 2017; Johansson and Nilsson 2002; Wang et al. 2016). Finally, other factors external to the construction of the RORs could have played a role. A series of large forest fires occurred across Quebec in May 2010, two years after construction. In particular, a large fire affected the area of Wemotaci (2010, Fig. 1A, D) causing the evacuation of 1300 residents. Details of the location of the burned area can be found on the map in Ponton et al. 2021 (Fig. 1 herein). We estimated the area burned in 2010 as being approximately 200 km^2^. This fire affected the eastern side of the Saint-Maurice River on a distance of 24.6 km upstream of CA ROR and continued for an additional 6.9 km downstream. Also, large areas around the Saint-Maurice River, particularly in the sector of RDC, are currently logged (Fig. 1C, D). We estimated that both sides of the river were heavily logged on a distance of 24.6 km of river stretch upstream of RDC ROR (Fig. 1D). Past studies have shown a link between such landscape disturbances and Hg cycling in lakes (Allen et al. 2005; Garcia et al. 2007; Skyllberg et al. 2009a). All these potential causes could have interacted with each other, and led to an increase in Hg methylation processes, leading to methylmercury bioaccumulation and biomagnification in the food web. Here, we first examine the response time of the system with respect to Hg fish contamination and then explore the different spatial changes in carbon sources and Hg biogeochemical transformation that occurred at the base of the food web and during trophic transfer.

### Magnitude of Increase and Recovery Time of Hg Levels in Fish from ROR Systems

**Fig. 3:**
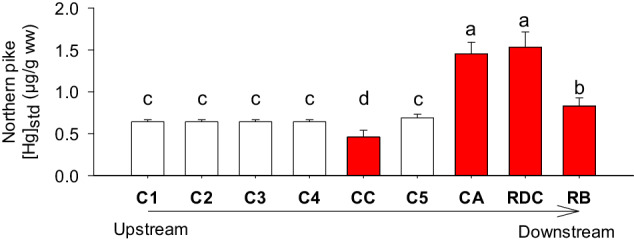
Spatial changes standardized Hg concentrations for northern pike (standardized at 600 mm) in red in the constructed channels (CC), Chute-Allard sampling sites (CA) and Rapides-des-Coeurs sampling sites (RDC), and the Reservoir Blanc (RB). The other control (C1 to C5, in white) sites are located upstream of CA ROR (see Fig. 1, C1 is a regional control value from multiple lakes; see Ponton et al. (2021) for details). Sites are positioned along an upstream-downstream sequence of the Saint-Maurice River stretch. Data are from 2016 (eight years after impoundment of CA and RDC RORs) (Cossette et al. 2019; Ponton et al. 2021; Sacotte et al. 2020). See Fig. 2 for details on standardization of Hg concentrations. The 95% confidence intervals (error bars) are presented; when these intervals do not overlap, there is a significant difference between sites (*p* < 0.05). Each polynomial regression (*n* = 30) to estimate [Hg_std_] respected assumptions of normality and homoscedasticity of the residuals

Regarding the concentration of Hg in predatory fish, five years after construction (i.e., in 2013), standardized Hg levels for 400 mm walleyes and 600 mm northern pike were approximately double the reference sites (Fig. 3 presented here) (Ponton et al. 2021). Specifically, northern pike and walleye standardized Hg concentrations in the run-of-river pondages were approximately 2.0 and 1.2 μg/g (wet weight, ww), respectively. These levels are above the Health Canada recommendations for commercially sold fish (0.5 μg/g ww (Health Canada 2007)). Subsequent surveys in 2016 and 2019 showed a decline in concentrations (Fig. 2A). Although Hg levels in 2019 fish have not yet reached pre-impoundment levels, they still indicate a relatively fast recovery of the system when compared to large reservoir systems, where levels peak on average 10 years after impoundment and may take 30 years to return to levels equivalent to those found in fish in surrounding waterbodies (baseline levels) depending on fish species (Bilodeau et al. 2017; Willacker et al. 2016). For these two RORs, the temporary increase of Hg in predatory fish peaked after *ca*. five years and returned to normal pre-dam levels within *ca*. 12 years. We compared these two post-impoundment response curves with the one obtained for pike from the nearby ROR built on the Péribonka River in a watershed with no recent disturbance (Fig. 2A; see Supplementary Table S1 for main characteristics of Péribonka vs RDC ROR and CA ROR). Although one can observe a small increase in Hg levels in pike in Péribonka after four to six years (up to 0.9 μg/g ww), this increase is about a third of what was observed in the disturbed watershed of CA and RDC RORs. It is therefore likely that the combined effects of watershed disturbances and the construction of dams led to a greater contamination of fish than dam construction alone.

### ROR Pondages as Main Sites of Hg Increase in Fish

To establish if these large fish Hg increases occurred throughout the Saint-Maurice River system or mainly in the CA and RDC ROR pondages, we compared fish caught in different river stretches (Fig. 3). Only sites with impoundments displayed an increase of Hg in fish. Of particular interest, fish caught in constructed channels were low in Hg, even lower than for reference unimpacted sites, contrary to expectations (Fig. 3).

Further, in some large reservoir dams, fish passing through the turbine are stunned and often severely impaired and become easy prey for fish downstream (Verdon et al. 1991). These fish found immediately downstream often become more piscivorous and have higher Hg levels. To assess such changes in RORs, we used data from the Péribonka dam, since no data are available directly downstream of CA and RDC RORs. Downstream pike and walleye were not more contaminated than those from the pondages (Fig. 2B). In RORs, the pressure difference between upstream and downstream is probably not high enough, and fish seem little affected by the transit through the ROR dam section.

### ROR-induced Site-dependent Changes at the Base of the Food Web

These discontinuities along the river were also apparent at the base of the food web. For instance, both dissolved MeHg concentrations and MeHg concentrations in periphytic biofilm mats that sustain part of the food web were enriched in the Chute-Allard area (Supplementary Fig. S1) (Leclerc 2022; Leclerc et al. 2023). Also, the proportion of MeHg over total Hg (a proxy of Hg methylation potential) in periphyton reached maximum values in flooded sites (*ca*. 23%), with values 2.9 times higher than in unflooded ones (Leclerc 2022; Leclerc et al. 2023). Further, flooding created scattered lake-like habitats which led to modifications in invertebrate trophic structures by the introduction of new carbon sources derived from littoral areas and bottom sediments (Leclerc et al. 2023).

**Fig. 4:**
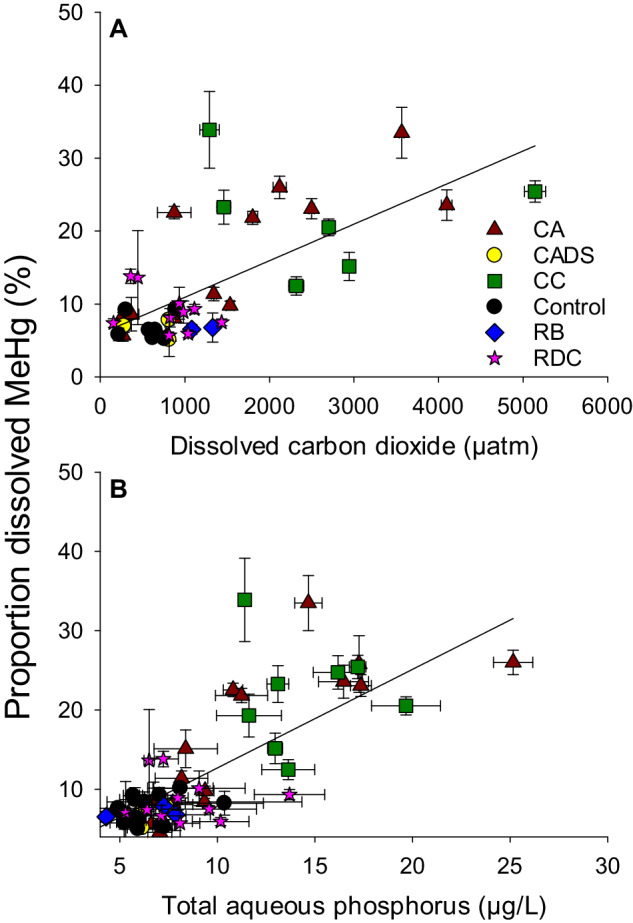
Proportions of dissolved MeHg (%) as a function of dissolved carbon dioxide concentrations (µatm, panel **A**; regression line is shown: R^2^ = 0.48; *p* < 0.001; *n* = 43), and total aqueous phosphorous (µg/L, panel **B**; regression line is shown: R^2^ = 0.60, *p* < 0.001; *n* = 60). River sectors legend is in panel **A**. Error bars refer to standard deviation. Original figure from data obtained during samplings and using methods described in (Ponton et al. 2021). Sampling site abbreviations: CA Chute-Allard, CADS Sector immediately downstream from the CA powerplant, CC constructed channels, RB Reservoir Blanc, RDC Rapide-des-Coeurs

Other chemical parameters in the water column for which discontinuities between constructed channels, pondages and reference sites were observed included dissolved carbon dioxide (CO_2_), methane (CH_4_, covariate with CO_2_), and total phosphorus (Supplementary Fig. S2). For instance, dissolved CO_2_ and CH_4_ concentrations were super-saturated in the channels and the pondages (Fig. 4), even 11 years after impoundment, indicating important microbial activity in these sectors. The constructed channels and bays of pondages have presumably longer residence times and lower turbulence than the main river channel, slowing the degassing of CO_2_ and CH_4_. These concentrations of greenhouse gases were highly correlated with the proportion of dissolved MeHg in water (Fig. 4A), indicating that the overall respiration of the system was linked to Hg methylation. This organic matter degradation was also related to a release of nutrients to the water column, leading to the observed relationship between phosphorus and %MeHg (Fig. 4B), especially in sampling sites near CA ROR (CA) and constructed channels (CC). It is likely that high methane, phosphorus and MeHg levels are related to the development of transient anoxic conditions. From these observations, we conclude that ROR construction and the associated flooding of even small area, caused a variety of physical, chemical and biological changes in the riverscape.

### Hg and MeHg Accumulation in Pondages Linked to Watershed Processes

**Fig. 5:**
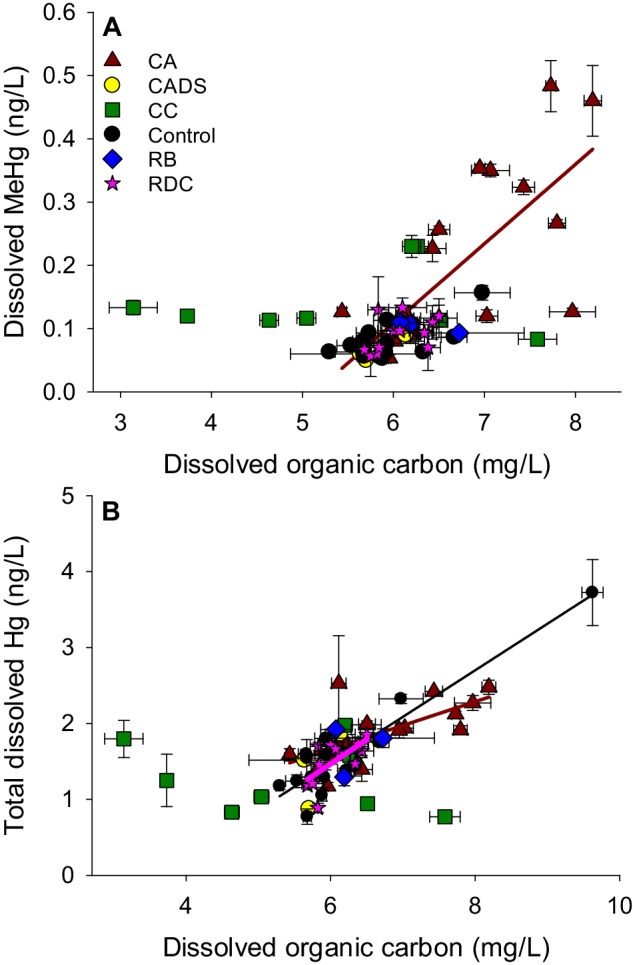
Dissolved MeHg (ng/L; panel **A**) and total dissolved Hg (ng/L; panel **B**) as a function of dissolved organic carbon concentrations (mg/L) per sector in the Saint-Maurice River. All years of data (2016–2019) are included. For panel **A**, regression line for Chute-Allard sites is shown: R^2^ = 0.54; *p* < 0.001; *n* = 19. For panel **B**, three regression lines are shown: for Rapide-des-Coeurs (RDC): R^2^ = 0.42; *p* = 0.02; *n* = 10; for Chute-Allard sites (CA): R^2^ = 0.45; *p* = 0.002; *n* = 17; for Control sites: R^2^ = 0.85; *p* < 0.001; *n* = 13. Error bars refer to standard deviation. Original figure from data obtained during samplings and using methods described in (Ponton et al. 2021). Sampling site abbreviations: CADS Sector immediately downstream from the CA powerplant, RB Reservoir Blanc, CC constructed channels

It is well recognized that carbon sources and Hg cycling are tightly linked in aquatic systems (Demarty et al. 2021). For instance, a relationship between dissolved organic carbon (DOC) and Hg in water is often encountered (Lavoie et al. 2019) at the landscape scale and is commonly attributed to the complexation of inorganic Hg with functional groups of dissolved organic matter (DOM). This DOM primarily originates from the degradation of organic matter in the watershed. Usually, the relationships between DOC and total Hg in water is stronger than the relationship between DOC and MeHg, since methylation entails additional steps within the aquatic system, causing a partial decoupling between variables (Lavoie et al. 2019). In the Saint-Maurice River, we observed clear ROR-induced discontinuities per sector between DOC and MeHg relationships (Fig. 5). The only significant relationship was found in the flooded sector of Chute-Allard, where 51% of the variance in MeHg was explained by DOC over the years. In the reference and RDC sectors, there was little variation in DOC concentrations compared to CA sites, which may explain the low correlation. In the constructed channels the widest range of DOC values was found, but with no link to MeHg. This may result from the fact that these channels did not receive Hg from the watershed via DOC-mediated transport, and that the DOC was probably formed within these newly constructed wetlands. Overall, it seems that the ROR-impacted CA sector accumulated Hg as a function of DOC inputs from the watershed, whereas this was not seen in other sectors.

Sediments were also followed along the ROR system, for Hg, MeHg, carbon/nitrogen (C/N) ratios and organic matter content (%OM). Highest concentrations of Hg, MeHg and %OM were found in the pondage areas (Ferriz et al. 2021). Concentrations of Hg and MeHg were best predicted by %OM, with 87 and 82% of the variance explained, respectively. The Hg methylation potential (%MeHg) was best explained by the C/N ratio in the sediments, with 87% of the variance explained (Ferriz et al. 2021). In the context of a river, sediment C/N ratios are indicative of the contribution of imports of organic carbon from the watershed, and/or from littoral algae or periphytic biofilms when light reaches the bottom. High C/N ratios are typically linked to degraded terrestrial biomass, rich in cellulose and poor in protein, whereas lower C/N ratios are linked to in-river primary production (e.g., algae, macrophytes). Taken together, these results suggest that pondages constitute areas where Hg transported by particles tend to accumulate in sediments, and where Hg methylation occurs, driven by co-transport of fresh organic matter from the watershed, in anoxic and reductive conditions partly due to the high bacterial respiration. This strong link between %MeHg and C/N ratios is unexpected, since recent evidence indicates that organic matter produced on site (e.g., by algae) tends to be more efficient at fueling microbial methylation (Bravo et al. 2017). In the case of the Saint-Maurice River, it is proposed that the recent watershed disturbances by wildfire (in the case of CA ROR) and logging (for RDC ROR) had a profound impact on organic carbon dynamics, mobilizing carbon and Hg from the landscape towards the river and to pondages created by dams where sedimentation was enhanced. Since low concentrations of MeHg were measured in the watershed soils, it is most likely that the MeHg found in sediments was produced in situ, and not transported as MeHg from the landscape.

With respect to the constructed channels, contrary to our hypothesis, concentrations of Hg and MeHg in sediments and water were low compared to other sites from the same river section (Ferriz et al. 2021). These channels were excavated, and the newly exposed sediments were initially sandy (reflecting Haute-Mauricie post glacial deposits) and gradually covered by autochthonous organic matter. The parent inorganic material was therefore low in organic matter and Hg, yielding low aqueous concentrations of Hg and MeHg (Fig. 5). The constructed channels did act as bioreactors of MeHg production since the %MeHg were somewhat elevated (Fig. 4), but this production was limited by low substrate levels (organic matter and inorganic Hg).

### Food Web Transfer and Link to Carbon/allochthonous Inputs

Carbon processing was also assessed to be of prime importance in determining the fate of Hg within the food web. First, %MeHg in the water column (influenced by microbial MeHg production in sediments and periphyton) was best explained by aqueous CO_2_ levels (Ponton et al. 2021) (Fig. 4 presented here). This suggests a link between rates of microbial mineralization of organic carbon and mercury methylation potential. Thereafter, MeHg bioaccumulated in lower trophic levels such as macroinvertebrates and benthivores. Their MeHg bioaccumulation were best explained by their δ^13^C signature, which was negatively correlated with MeHg (Ponton et al. 2021). This implies a link between ecosystem organic carbon degradation and MeHg accumulation. This was somewhat surprising, since few studies in rivers have linked MeHg bioaccumulation to δ^13^C in tissues (Riva-Murray et al. 2013).

Higher in the food web, Hg levels were well predicted by the nitrogen isotopic signature (δ ^15^N, a proxy of trophic position). However, the best prediction model of MeHg food web transfer combined both the δ^15^N and δ^13^C signatures, again stressing the importance of organic carbon transformation and cycling (Ponton et al. 2021). Further, the study by Ponton et al. (2021) reported the slope of the relationship between trophic position (using δ^15^N as a proxy) and the log-transformed concentration of Hg animal species, the so-called trophic magnification slope (TMS) (for information on TMS, see (Lavoie et al. 2013), which is used to compare the efficiency of a food web to transfer contaminants. These TMS did not show any upstream-downstream trends in this study, indicating that damming and other landscape disturbances did not alter mercury food web dynamics with respect to efficiency of transfer (Ponton et al. 2021). However, the trophic magnification intercepts of this relationship did change with sectors and could be a relevant variable to follow in the future, instead of focusing on TMS. Indeed, this intercept may be a good proxy of the MeHg bioaccumulation at the base of the food web.

With respect to constructed channels, there was a production of MeHg in periphytic biofilms followed by a transfer to macroinvertebrates (Leclerc et al. 2021; Leclerc et al. 2023), but this transfer did not lead to increased Hg levels in fish (Fig. 3). We hypothesize that the high microbial respiration in CC (Fig. 4A) may have led to periods of hypoxia that prevented fish from significantly feeding in the area.

### Main Microbial Hg Methylators

About a decade ago, it was discovered that Hg microbial methylation requires the gene pair hgcAB, which encodes for proteins that actuate Hg methylation (Parks et al. 2013). In the Saint-Maurice River system, metagenomic analysis was performed on Hg-methylating microbial communities living in sediments and in photosynthetic biofilms developing on wood debris and aquatic plants (macrophytes and periphyton), based on the hgcA functional gene marker (Ferriz et al. 2021; Leclerc et al. 2021). In sediments, this analysis indicated an abundance of methanogens, fermenters and sulfate reducers, suggesting that these metabolic guilds may be important Hg methylators in surface sediments of the Saint-Maurice River (Ferriz et al. 2021). More recently, a genome catalog of Hg methylating microorganisms was created and a unique and diverse assemblage of Hg methylators emerged from 2018 Saint-Maurice River sediment samples, dominated by members of metabolically versatile and fermentative *Bacteroidota* (Lawruck-Desjardins 2023). This assemblage was particularly enriched in butyrate fermentation, carbon-fixing and potentially nitrite-reducing microbes. We found that sites affected by continuous long-term disturbances such as logging were particularly favorable to the establishment of a Hg methylating niche. Lastly, it was argued that the effects of watershed disturbances are likely not specific to Hg methylators, but rather shared across the greater microbial community (Lawruck-Desjardins 2023).

In periphyton, in addition to these guilds, iron reducers were also important (Leclerc et al. 2021). These results clearly indicate that both sediments and periphytic biofilm microbial communities have the required genes to methylate Hg and that watershed disturbances likely promote microbial activity in general, and the vast majority of Hg methylators.

## Comparison with Other Studies

Few studies have specifically assessed the effect of ROR on Hg cycling. The main one done at a comparable latitude to the Saint-Maurice study is the one conducted by Silverthorn et al. on seven regulated (capacity ranging from 7.6 to 49.9 MW) and six free-flowing mountain streams in coastal British Columbia, Canada (Silverthorn et al. 2018). Their main conclusion was that, on average, Hg concentrations in the blood and feathers of a predatory river-resident passerine was not significantly different between types of streams. However, in one case of a recently regulated stream (five years after dam commission), the authors reported Hg concentrations of potential toxic concern for the passerine, and partly attributed it to enhanced in situ MeHg production in the regulated stream. They also emphasized that elevated regional Hg deposition rates could be a significant factor for their densely forested Coastal Mountain stream systems. The maximum bird Hg concentration for this regulated system was found in the pondages rather than at sites upstream or downstream. These results indicate that RORs do not always cause increases in MeHg accumulation up the food web and that many factors must be considered, such as changes in food web dynamics, stream geochemistry or watershed disturbances. The authors recommended monitoring Hg concentrations and isotopic profiles of the entire food web before and after run-of-river regulation, upstream and downstream of the intake. They also indicated that to prevent MeHg production within the pondage area, it is recommended to screen sites for levels of atmospherically deposited Hg and maintain adequate flow within pondages to prevent anaerobic conditions.

To our knowledge, the only other published study specifically on Hg cycling and food web accumulation in rivers impacted by small hydropower plants centers on two tropical dammed systems part of the Upper Guaporé River basin floodplain of Brazil (Cebalho et al. 2017) (Cabixi power plants; 2.7 MW). This study mainly focused on following Hg levels in fish located in the pondages and downstream from them. The authors reported low Hg concentrations that did not exceed the safety limit for fish consumption recommended by the World Health Organization (WHO; 0.5 ppm). In general, they found higher Hg levels in fish from pondages than those collected downstream. However, since fish size for a given species varied between sectors and since Hg levels were not standardized for a given length, it is difficult to draw a clear conclusion. The authors partly attributed the low Hg levels found in fish compared to other dammed systems in Brazil to high oxygen water concentrations, low Hg methylation after flooding and the absence of thermal stratification.

Other studies have been conducted on the interactions of RORs with the biogeochemistry, ecology, sediment, and landscape processes of the system, but did not include Hg in fish in their analyses. For instance, Sow et al. (2016) performed a year-round survey of nutrients, suspended particulate matter and phytoplankton in a ROR with a hydraulic residence time (HRT) of a few days, and showed that the pondage retained 24% of soluble reactive phosphorus, this retention occurring mostly during the summer months. They call for the need to consider the impact of “minor” hydrological discontinuities such as RORs on the functioning of the river continuum.

Further, a recent paper by Ma et al. (Ma et al. 2023) investigated the impacts of river damming on sediment methylmercury dynamics in two Chinese rivers. While not specifically targeting ROR, the range of HRT considered included those found in CA ROR (0.002 year) and RDC ROR (0.006 year), on the lower side of their ranges (HRT range: 0.002–2.36 years). They concluded that river damming resulted in the accumulation of sediment Hg and organic matter and a higher abundance of *hgc*AB genes, causing an increase in %MeHg. They found that %MeHg was positively correlated to HRT. However, their sediment %MeHg were all between 0 and 0.6%, *ca*. an order of magnitude lower than what we observed in the Saint-Maurice (up to 10%) (Ferriz et al. 2021). We propose that the organic inputs to the Saint-Maurice caused by other watershed disturbances may lead to conditions promoting Hg methylation in this river.

## Emerging Conceptual Model

**Fig. 6:**
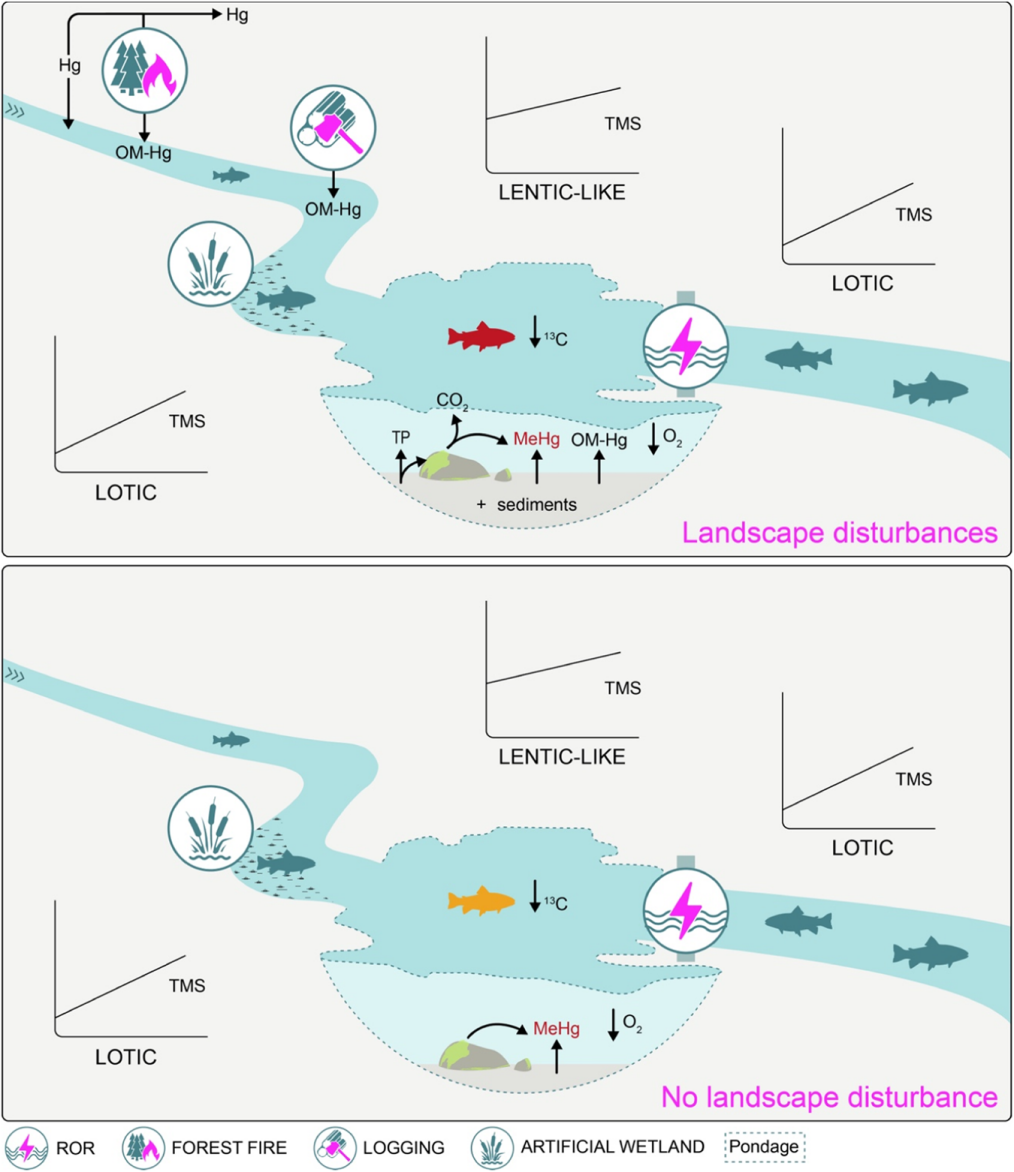
Conceptual model representing the impact of ROR with or without landscape disturbances on several ecosystem characteristics. TP total phosphorus, OM-Hg complex between organic matter and Hg

Our multi-year study encompassing all food web levels from bacteria to fish and including main Hg methylating matrices (periphyton and sediments) allows us to derive some conclusions that can be integrated in an initial conceptual model for rivers harnessed with RORs in North American environments, in the presence or absence of watershed disturbances (Fig. 6). We propose that even at very low HRT (in the range of hours to days), significant retention of Hg exported from disturbed watersheds can occur in ROR pondages. Further, even with limited flooding in the range of a few km^2^, flooded environments conducive to Hg methylation can emerge and cause pondages to become hotspots of MeHg production.

In cases where the watershed is not disturbed (Fig. 6, bottom panel), ROR dams will retain some particulate matter and some flooded environments may experience low oxygen conditions conducive to Hg methylation in sediments and periphyton. The food web may be locally modified into more lentic conditions and the intercept of the TMS may be increased. As a result, top predators may experience a small temporary increase in Hg levels, as was observed at the Péribonka ROR discussed above (Fig. 2).

When landscape disturbances such as logging or forest fires are present (Fig. 6, top panel), additional carbon and Hg inputs to the river may occur. For instance, forest fires will release Hg to the atmosphere that may be deposited locally to the river (Li et al. 2022). Further, the removal of vegetation by fire will allow a more efficient transfer of soil carbon and Hg by runoff to the river during rain events (Caldwell et al. 2000; Kelly et al. 2006). This latter phenomenon will also take place when vegetation is removed by logging and associated activities such as site preparation (Eklöf et al. 2014; Skyllberg et al. 2009b). This added Hg and organic carbon will be partly retained by the ROR dams (Todorova et al. 2016). The additional allochthonous organic carbon inputs will increase microbial respiration (higher CO_2_ and CH_4_ levels), causing more oxygen-depleted environments, a heightened production of MeHg by microbial methylators and a release of phosphorus in the water (Maavara et al. 2020). Intercepts of TMS (but not their slope) will be higher than in undisturbed systems, leading to high Hg levels in top predators, sometimes exceeding health guidelines (Ponton et al. 2021). Food webs from pondages will have a more negative δ^13^C signature.

In contrast to large power plants where fish are more contaminated downstream from dams (because fish passing through large dam turbines can be disoriented or killed, becoming easy prey for downstream fish), ROR may cause Hg increase mostly in pondages (Fig. 6). The combined effects of watershed disturbances and RORs are likely most felt in smaller rivers which are more influenced by allochthonous inputs (Doretto et al. 2020). These perturbed environments can harbor a community of Hg methylating microorganisms that is much more diverse than previously thought (Lawruck-Desjardins 2023). Specific taxonomic groups such as *Bacteroidota* and guilds such as butyrate fermenters, carbon fixing and nitrite reducing microbes are emerging as new players (Lawruck-Desjardins 2023).

However, the increase of Hg levels in food webs and in fish in particular is temporary and the return to pre-impoundment conditions can be *ca*. half what is commonly observed in large northern reservoirs (here, 10–15 years, compared to a mean of 30 years for large reservoirs (Bilodeau et al. 2017; Willacker et al. 2016)).

## Recommendations

This case study has shown the potential of ROR pondages to contribute to an unexpected temporary increase in mercury levels in food webs by Hg. As a result, we recommend that future projects considering RORs should include a monitoring component with surveys before and after construction including the downstream sector of the RORs (Smokorowski and Randall 2017). Efforts should focus on fish which are the main source of human exposure to mercury. An emphasis on monitoring pondages is particularly warranted. Such monitoring is important, since very few data exist on Hg cycling in ROR projects, and the present case study should be seen as a starting point. The follow-up period could be in the range of 20 years, considering that the response curve found here is about 12 years. This monitoring period should be adjusted according to the follow-up results. An adaptative monitoring plan should set out ahead of time when sampling should be increased or decreased (Somers et al. 2018). Further, since watershed disturbances can in some cases (such as logging) interact with ROR construction to create MeHg hotspots, potential landscape disturbances upstream of ROR projects should be considered in the choice of ROR locations.

Additionally, based on lessons learned from studies focusing on reservoirs (Bilodeau et al. 2017; Lucotte et al. 1999), variations observed in Hg levels in fish can be explained by physical and hydrological characteristics such as land characteristics of flooded areas, water residence time, duration of the impoundment period, water temperature, and percentage of flooded area located in drawdown zones. When developing an ROR facility, (i) flooding of soils should be minimized to reduce the quantity of organic matter that may stimulate bacterial methylation of inorganic Hg and (ii) the annual water volume passing to the ROR should be relatively high to dilute the Hg release in the water column and to ensure good water oxygenation thereby reducing methylation.

With respect to the creation of constructed wetlands as a remedial measure for communities, our results do not conclusively show an impact of such wetlands on Hg fish contamination. This is likely because these wetlands were constructed by excavation and the resulting sandy sediments were low in Hg and organic matter. We therefore recommend that future constructed wetlands should be built in such a manner. Still, constructed wetlands positioned upstream from RORs should be monitored as potential sources of MeHg.

Finally, it is recommended that a communication program be put in place, in collaboration with public health agencies, in order to address the issue of mercury with local communities. Co-partnerships between utilities, academics and communities is encouraged in order to foster trust when disseminating results, and to empower communities (Gérin-Lajoie et al. 2018). Messages and actions must be adapted according to each cultural realities and the benefits of consuming fish must be highlighted despite the presence of mercury. This is particularly true in cases where food security is an issue and when access to high quality market food is difficult. A risk/benefit approach is therefore proposed, whereby nutrients (fatty acids, proteins, selenium, iron, calcium, etc.) are measured in addition to mercury (MacMillan et al. 2022; Ponton et al. 2022).

## Supplementary information

Supplementary information
