# Supplementary material for: Cumulative Effects of Watershed Disturbances and Run-of-river Dams on Mercury Cycling: Case Study and Recommendations for Environmental Managers
Source: Environ Manage. 2024 May 22;75(11):3092–103. doi: 10.1007/s00267-024-01990-6 (PMC12546387; doi:10.1007/s00267-024-01990-6)
Supplement: Supplementary file 1 — Supplementary information [file 267_2024_1990_MOESM1_ESM.docx]

**Supplementary information**

**Cumulative effects of watershed disturbances and run-over-river dams on mercury cycling: case study and recommendations for environmental managers**

M. Amyot^1*^, F. Bilodeau^2^, A. Tremblay^2^, D. Planas^3^, D. Walsh^4^, D.E. Ponton^1^

^1^ *GRIL, Département de Sciences Biologiques, Université de Montréal, 1375 Thérèse-Lavoie-Roux Ave. Montréal, QC H2V 0B3, Canada.*

^2^ *Hydro-Québec, Direction Environnement, 800 De Maisonneuve Est Blvd., Montréal, QC H2Z 1A4, Canada.*

^3^ *GRIL, GEOTOP, Département de Sciences Biologiques, Université du Québec à Montréal, 141 Président-Kennedy Ave., Montréal, QC H2X 1Y4, Canada.*

^4^ *GRIL, Department of Biology, Concordia University, 7141 Sherbrooke St. West, Montréal, QC H4B 1R6, Canada.*

* Corresponding author: m.amyot@umontreal.ca

**Details of fish sampling**

**Fish Sampling.** In August 2013, 2016, and 2019 (5-, 8-, and 11-years post-impoundment of CA and RDC), a private consulting firm (AECOM, Trois-Rivières, QC, Canada) collected 584, 1404 fish, and 1314 fish, respectively. The experimental fishing was carried out using experimental Grand Nord type gillnets measuring 61 m in length and 2.4 m in height. These nets are composed of eight panels, each 7.6 m in length, with stretched meshes of 25, 38, 51, 64, 76, 102, 127, and 152 mm, respectively. These fish were collected to evaluate the influence of ROR on the fish community. AECOM also sampled muscle tissue collected close to the dorsal fin from 32 individuals to analyze [Hg]. The collected species were yellow perch (*Perca flavescens*), walleye (*Sander vitreus*), northern pike (*Esox lucius*), longnose sucker (*Catostomus catostomus*), white sucker (*Catostomus commersonii*), lake whitefish (*C*oregonus clupeaformis), fallfish (Semotilus corporalis), and common shiner (Luxilus cornutus). Fish weight and length were measured. The stomach contents were weighted, sorted and identified.

**Table S1.** Comparison of main characteristics of four hydroelectric powerplants.


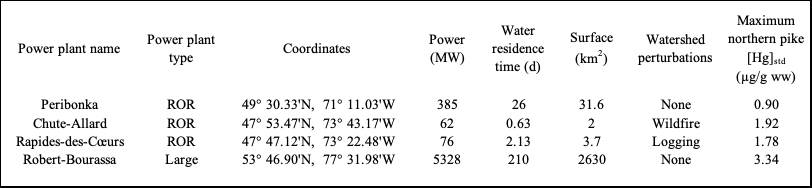


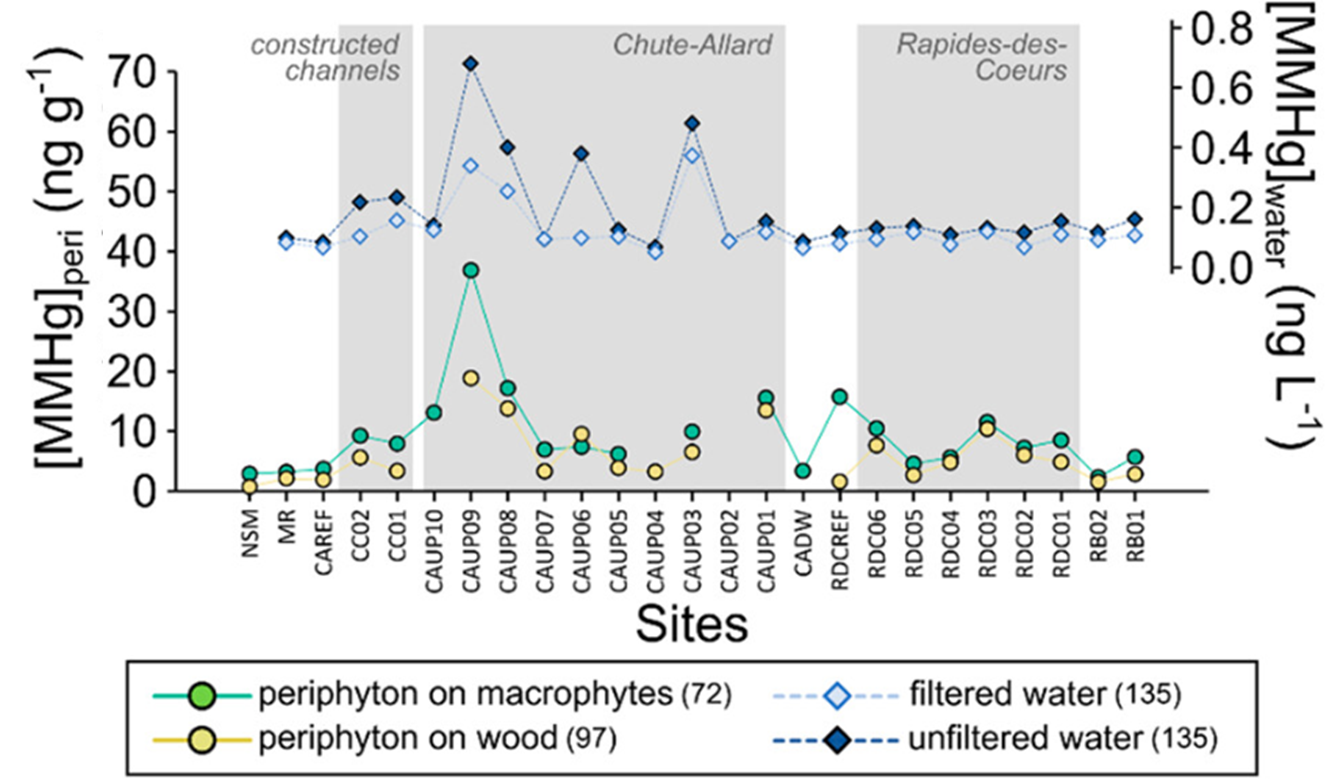


**Figure S1.** Concentrations of MeHg in periphyton and water along an upstream/downstream transect affected by ROR construction (Chute-Allard and Rapide-des-Coeurs power plants) on the Saint-Maurice River. Adapted from Leclerc et al. (2023)^1^.


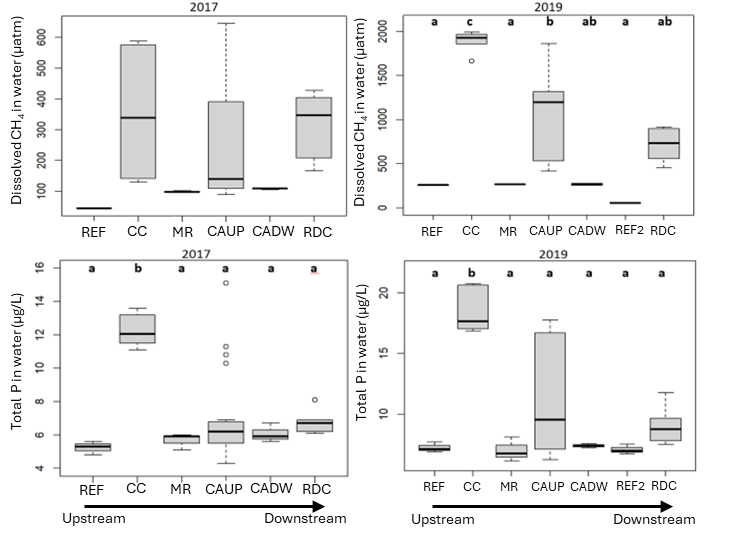


Fig. S2. Spatial changes in methane (CH_4_, µatm) and total phosphorus (TP, µg/L) along a transect of the St. Maurice River affected by RORs. Edges of boxes represent 25th and 75th percentiles, whiskers represent 95^th^ percentiles and the lines in the boxes represent medians. Results of a Tukey test are presented with letter on top of graphs. This information has not been presented elsewhere in this format but see details of methods and other uses of the data set in ^2^.

**References**

1. Leclerc, M.; Ponton, D. E.; Bilodeau, F.; Planas, D.; Amyot, M., Enhanced Bioaccumulation and Transfer of Monomethylmercury through Periphytic Biofilms in Benthic Food Webs of a River Affected by Run-of-River Dams. *Environ Sci Technol* **2023,** *57*, (49), 20792-20801.

2. Ponton, D. E.; Lavoie, R. A.; Leclerc, M.; Bilodeau, F.; Planas, D.; Amyot, M., Understanding Food Web Mercury Accumulation Through Trophic Transfer and Carbon Processing along a River Affected by Recent Run-of-river Dams. *Environ Sci Technol* **2021,** *55*, (5), 2949-2959.
